# Three-Headed Biceps Brachii Muscle: A Rare Site of Proximal Median Nerve Entrapment

**DOI:** 10.7759/cureus.61886

**Published:** 2024-06-07

**Authors:** Randy Kulesza, Caitlin Sachsenmeier, Faith Klein

**Affiliations:** 1 Anatomy, Lake Erie College of Osteopathic Medicine, Erie, USA

**Keywords:** biceps brachii muscle, orthopedics, muscles, nervous system, dissection, anatomy

## Abstract

The biceps brachii muscle is a highly variable muscle in the anterior compartment of the arm, and the most common variants include additional heads or slips. The median nerve courses with the brachial artery in the medial arm near the biceps brachii muscle, crosses the elbow, and enters the forearm deep to the bicipital aponeurosis. While entrapment of the median nerve in the carpal tunnel is one of the most common neuropathies, more proximal entrapments by the bicipital aponeurosis or other variants have been reported. In a 94-year-old embalmed female cadaver received through the Humanity Gift Registry of Pennsylvania, a biceps brachii muscle with an additional slip that arose from the coracoid process was found, which bridged over the median nerve and blended with the investing fascia of the forearm flexors via aponeurosis. Because of the course of this muscular slip in the arm and its relationship to the median nerve, this may be an additional site of proximal entrapment of the median nerve. It is important to consider these rare sites of nerve entrapment when diagnosing patients with median nerve neuropathy.

## Introduction

The biceps brachii muscle is situated in the anterior compartment of the arm and arises from two proximal attachments. The long head is situated laterally and arises from the supraglenoid tubercle of the scapula, and the medially situated short head arises from the coracoid process. The two heads of the biceps brachii converge to form a single common tendon that attaches to the radial tuberosity. Additionally, there are aponeurotic fibers derived from the tendon, termed “bicipital aponeurosis” (BA) (lacertus fibrosus [1]), that extends medially and inferiorly to blend with the antebrachial fascia over the superficial layer of forearm flexors. The biceps brachii is innervated by the musculocutaneous nerve, and the main actions are supination and flexion of the elbow, with the short head of biceps brachii aiding in adduction and flexion of the shoulder joint [2].

The biceps brachii muscle is highly variable; accessory muscular heads or slips occur in 8-23% of individuals [3,4]. Although accessory heads have been reported to arise from the deltoid tuberosity, pectoralis major, and teres major, the most common accessory slips occur along the medial side of the muscle [5]. These common accessory slips arise from the humerus, coracobrachialis, or brachialis muscles [5], may extend only to the humerus or brachialis muscle, or may extend across the elbow to blend with the BA or antebrachial fascia or may even attach to the radius or ulna. Accessory heads can also arise in common with the short head from the coracoid process and extend to the medial intermuscular septum or medial epicondyle [6,7]. Such a slip has been observed to entrap the brachial artery [6,7]. A muscular slip arising from the short head, crossing the brachial artery, and inserting along the fascia of the pronator teres has been identified (brachio-fascialis [5]). Additionally, the BA may be doubled and compress the brachial artery and/or median nerve in the cubital fossa [8].

The median nerve is formed from the lateral and medial cords of the brachial plexus and typically includes axons from C5-T1 levels of the spinal cord as well as post-ganglionic sympathetic axons from T2-T7 [2]. The median nerve courses medial to the short head of the biceps brachii with the brachial artery, passes deep to the BA, commonly courses between the two heads of the pronator teres, and then courses deep to flexor digitorum superficialis toward the carpal tunnel. While the carpal tunnel is the most common site of entrapment of the median nerve, there are several reports of more proximal entrapments of this nerve due to its relationship with the BA and flexor digitorum superficialis, but also from anatomical variants in the distal arm such as the ligament of Struthers [7,9,10]. Herein, we report a rare variant of the biceps brachii muscle that bridged over the median nerve and brachial artery, forming a rare supracondylar entrapment of these structures.

## Case presentation

**Figure 1:**
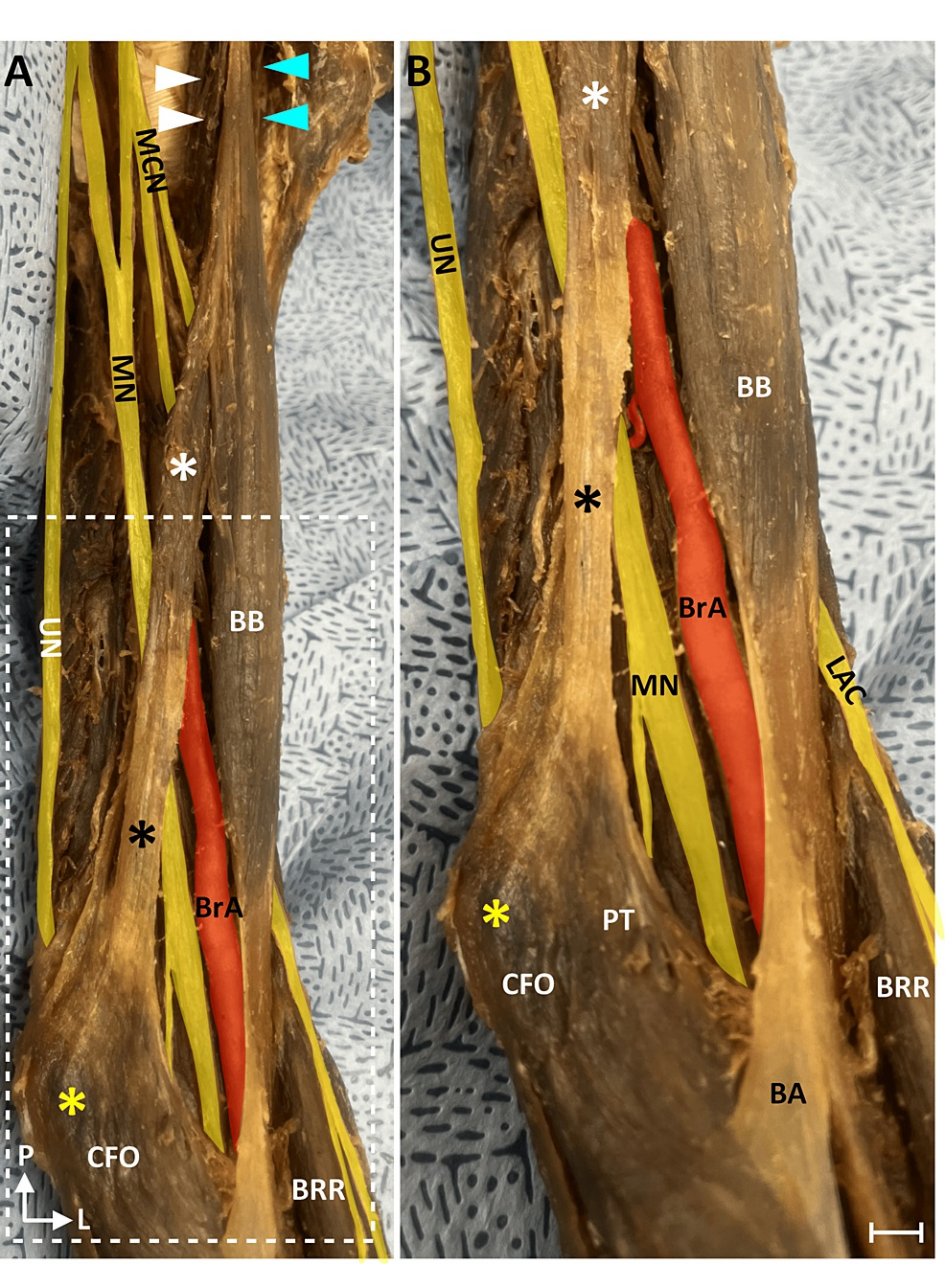
Accessory slip of the biceps brachii muscle The left arm from the dissected specimen is shown from the medial side (A). The region indicated by the white rectangle in A is shown magnified in B. Nerves are colored yellow, and the BrA is red. The coracobrachialis had a single head (white arrowheads). The long head of the BB is indicated by cyan arrowheads. The accessory slip is indicated by the white asterisk, and its aponeurosis is indicated by a black asterisk. The accessory muscle slip passed medial to the median nerve and BrA. The aponeurosis of the accessory slip blended with the PT and the CFO. The medial epicondyle is indicated by the yellow asterisk. Scale bar is equal to 4 mm. BA, bicipital aponeurosis; BB, biceps brachii muscle; BrA, brachial artery; BRR, brachioradialis muscle; CFO, common flexor origin; L, lateral; LAC, lateral antebrachial cutaneous nerve; MN, median nerve; MCN, musculocutaneous nerve; P, proximal; PT, pronator teres; UN, ulnar nerve

The variant discussed below was identified in an embalmed cadaver of a 94-year-old female (cause of death: failure to thrive) during a routine dissection within the Human Gross Anatomy course at the Lake Erie College of Osteopathic Medicine. The dissection was completed utilizing standard guidelines. The cadaver was received from the Humanity Gifts Registry of Pennsylvania. We identified a biceps brachii muscle with an additional slip along the medial aspect of the muscle in the left arm only (Figure 1).

This additional slip arose in common with the short head from the coracoid process. This slip split from the short head in the proximal arm and descended medially toward the medial epicondyle. This additional slip formed an aponeurosis (Figure 1) rather than a tendon, which blended with the investing fascia of the common forearm flexors. This additional slip and its aponeurosis appeared as a more proximal slip of the BA. The muscle belly was situated anterior to the median nerve and brachial artery. The median nerve passed under the muscle at its aponeurosis 8 cm proximal to the BA. This distance was measured with a standard ruler. This additional slip of the biceps brachii muscle provided only a very narrow tunnel for the median nerve and brachial artery and therefore may form a rare site of supracondylar median nerve entrapment.

## Discussion

On day 24 of embryological development, the upper limb bud appears at the level of C5 to T1. During week 5, myoblasts from the hypaxial dermomyotome invade the developing limb bud. Specifically, myoblasts from the dorsal muscle mass form the anterior compartment of the arm [11]. Myogenesis occurs in three waves: primary myogenesis in the embryo, secondary myogenesis in the fetus, and postnatal muscle growth in response to mechanical stimuli or muscle damage [11]. Mehta et al. noted that proper distribution of myoblasts throughout the developing limb bud depends on intricate interactions between growth factors expressed by cells in the limb bud and adhesion molecules expressed by myoblasts, and any disruption of this process may lead to accessory muscles or attachments [12]. These accessory muscles or attachments can lead to other sites of entrapment neuropathies, like those described throughout the rest of this discussion.

**Figure 2:**
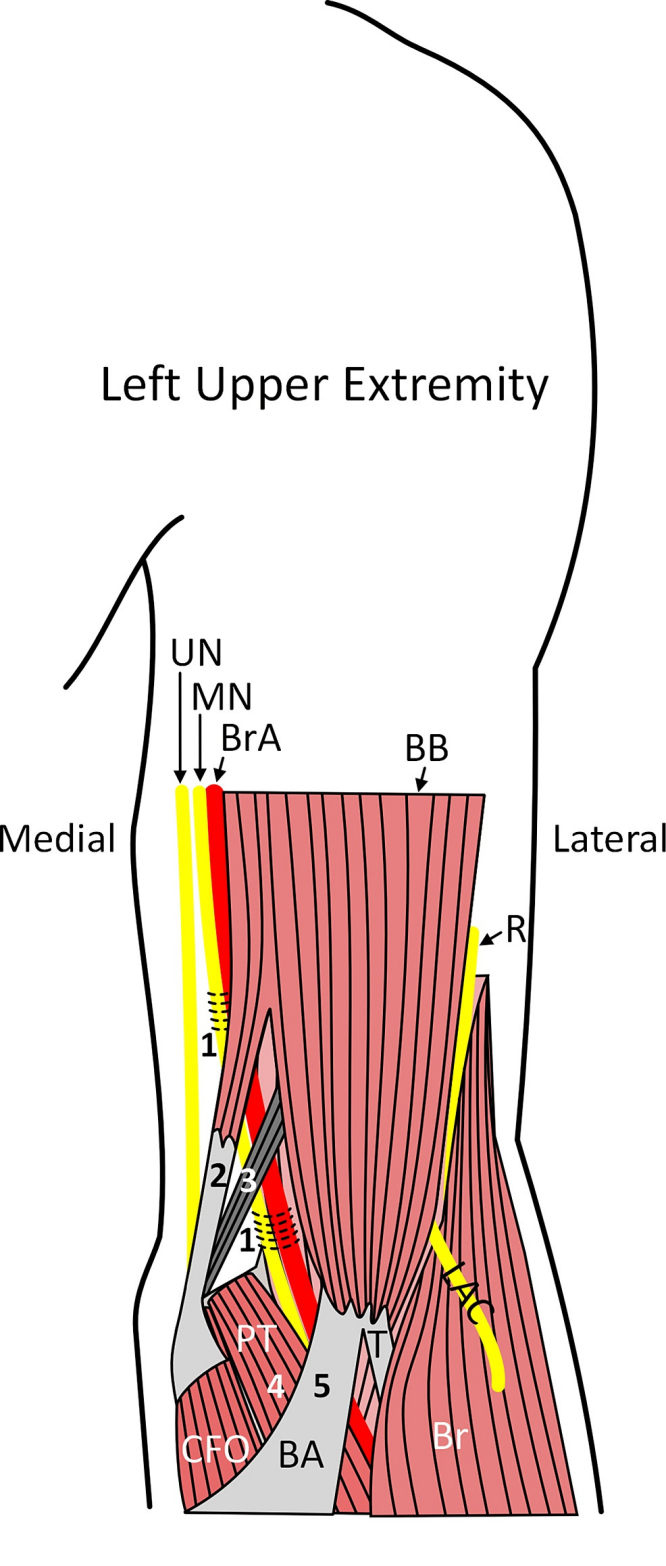
Summary of etiologies for proximal entrapments of the median nerve Nerves are shown in yellow, and the brachial artery is shown in red. The horizontal lines indicate sites of fascial entrapment of the median nerve. The numbers correspond to reported sites of median nerve entrapment. The median nerve can be entrapped or compressed by fascial tunnels or thickening of the deep brachial fascia (#1), muscular variants that attach to the medial epicondyle or deep fascia (#2) such as an accessory slip of the biceps brachii, or the ligament of Struthers (#3). Distal to the elbow, the median nerve can be entrapped by the pronator teres (#4) or BA (#5). BA, bicipital aponeurosis; BB, biceps brachii muscle; Br, brachioradialis; BrA, brachial artery; CFO, common flexor origin; LAC, lateral antebrachial cutaneous nerve; MN, median nerve; PT, pronator teres; R, radial nerve; UN, ulnar nerve Image credits: Kulesza R

Compression of the median nerve in the carpal tunnel is one of the most common entrapment neuropathies, but there are also several reports on median nerve entrapment in the proximal forearm by the ligament of Struthers, BA, pronator teres, or flexor digitorum superficialis, and variations associated with this muscle (i.e., Gantzer’s muscle [13]). There are also several reports of supracondylar median nerve entrapments (i.e., proximal to the elbow) [1,7-9,14-16] within musculofascial tunnels related to the coracobrachialis, brachialis, biceps brachii, deep muscular fascia, or intermuscular septum. These proximal entrapments are summarized in Figure 2. One such muscular variant was found arising from the coracoid process and attaching to the humerus [15,17] and was termed “corachobrachialis longus” or Wood’s muscle. Piyawinijwong et al. [18] described two cases of proximal MN entrapment from a cadaveric study. Their first case involved a thickening of the brachial fascia that attached to the supracondylar ridge and formed a tunnel for both the median nerve and brachial artery (Figure 2) [18]. Their second case involved a “tiny” muscular slip from the short head of biceps brachii that joined the BA, resulting in a thickening of the aponeurosis that compressed the median nerve [16].

Three cases of proximal median nerve entrapment by abnormal muscles in the arm were described by Paraskevas et al. [19]. They identified a slip from the tendon of the long head of biceps and coracobrachialis that inserted along the intermuscular septum and brachial fascia and formed a tunnel for both the median nerve and ulnar nerve (Figure 2) [19]. They also found a muscular slip arising from the medial aspect of the humerus that inserted onto the biceps brachii and had fibers joining the intermuscular septum that formed a tunnel for the median nerve and brachial artery. Finally, they identified a muscular slip arising from the brachialis muscle, arching over the median nerve and brachial artery before blending into the intermuscular septum [19]. While these cases of supracondylar median nerve entrapment are from cadaveric studies, there is also a report of a 44-year-old-male who presented with sensory loss on the palmar aspect of his thumb, index finger, and middle finger, consistent with median nerve injury [7]. This patient had a negative Tinel's sign at the wrist but a positive Tinel’s sign superior and medial to the medial epicondyle - together suggesting entrapment in the proximal arm. The patient’s deficits were resolved with the division of a fascial tunnel in the medial arm. Because these more proximal entrapments are relatively infrequent and present with more extensive weakness (weakness in wrist and finger flexion) and sensory disturbances than carpal tunnel syndrome, a detailed knowledge and understanding of the course of the median nerve as well as associated anatomical variants along the course of the nerve is needed. Accordingly, musculofascial variants should be considered in atypical cases of median nerve entrapment.

## Conclusions

The most common cause of median nerve neuropathy is compression within the carpal tunnel. While more proximal compressions are less common, they can be difficult to diagnose because they are encountered so infrequently and therefore less well documented and variable. More proximal compressions or entrapments of the median nerve may occur distal to the elbow and are caused by the nerve’s relationship to normal muscles or their variants. These more proximal compressions are significant because they can result in sensory changes and weakness along the entire distribution of the median nerve.

This case report describes a variant of the biceps brachii with attachment to the medial epicondyle of the humerus that formed a bridge over the median nerve and brachial artery, and this narrow canal provides a potential site of compression of the median nerve, proximal to the elbow. Compression at this location should be easily distinguished from entrapment in the carpal tunnel but may appear very similar in terms of signs and symptoms of more proximal nerve entrapments by musculofascial structures. Nonetheless, clinicians should be aware of the possibility of variants of the biceps brachii in proximal compressions of the median nerve, as this would change the management of signs and symptoms or procedures to release the nerve.
